# Supplementary material for: CyAbrB2 is a nucleoid-associated protein in Synechocystis controlling hydrogenase expression during fermentation
Source: eLife. 2024 Sep 2;13:RP94245. doi: 10.7554/eLife.94245 (PMC11368403; doi:10.7554/eLife.94245)
Supplement: Source data 1. [file elife-94245-data1.zip › original_images/Figure3-figure supplement 3.pptx]

## Slide 1
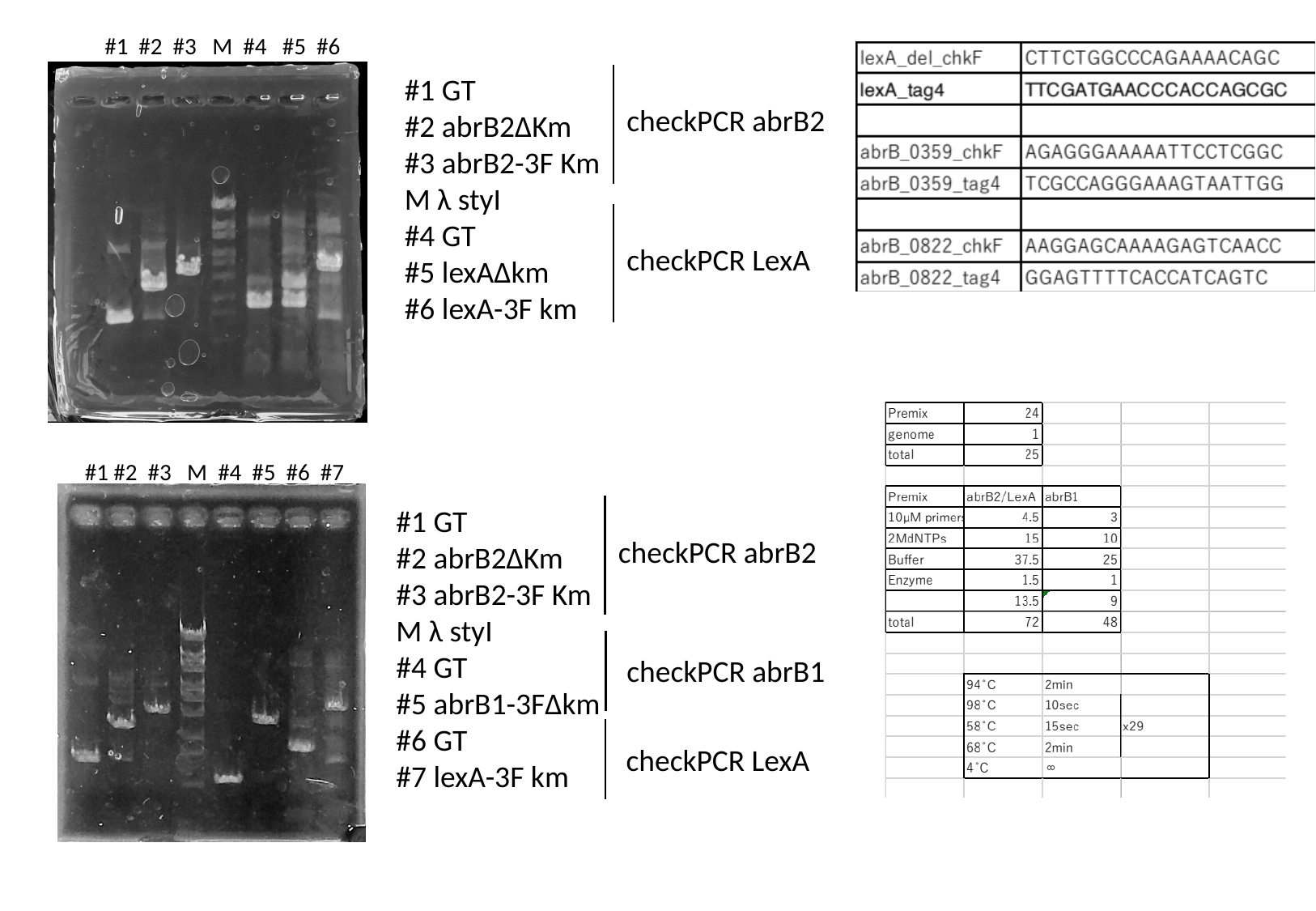

#1 #2 #3 M #4 #5 #6
#1 GT
#2 abrB2∆Km
#3 abrB2-3F Km
M λ styI
#4 GT
#5 lexA∆km
#6 lexA-3F km
checkPCR abrB2
checkPCR LexA
#1 #2 #3 M #4 #5 #6 #7
#1 GT
#2 abrB2∆Km
#3 abrB2-3F Km
M λ styI
#4 GT
#5 abrB1-3F∆km
#6 GT
#7 lexA-3F km
checkPCR abrB2
checkPCR abrB1
checkPCR LexA
