# Supplementary figures and images for: CyAbrB2 is a nucleoid-associated protein in Synechocystis controlling hydrogenase expression during fermentation
Source: eLife. 2024 Sep 2;13:RP94245. doi: 10.7554/eLife.94245 (PMC11368403; doi:10.7554/eLife.94245)

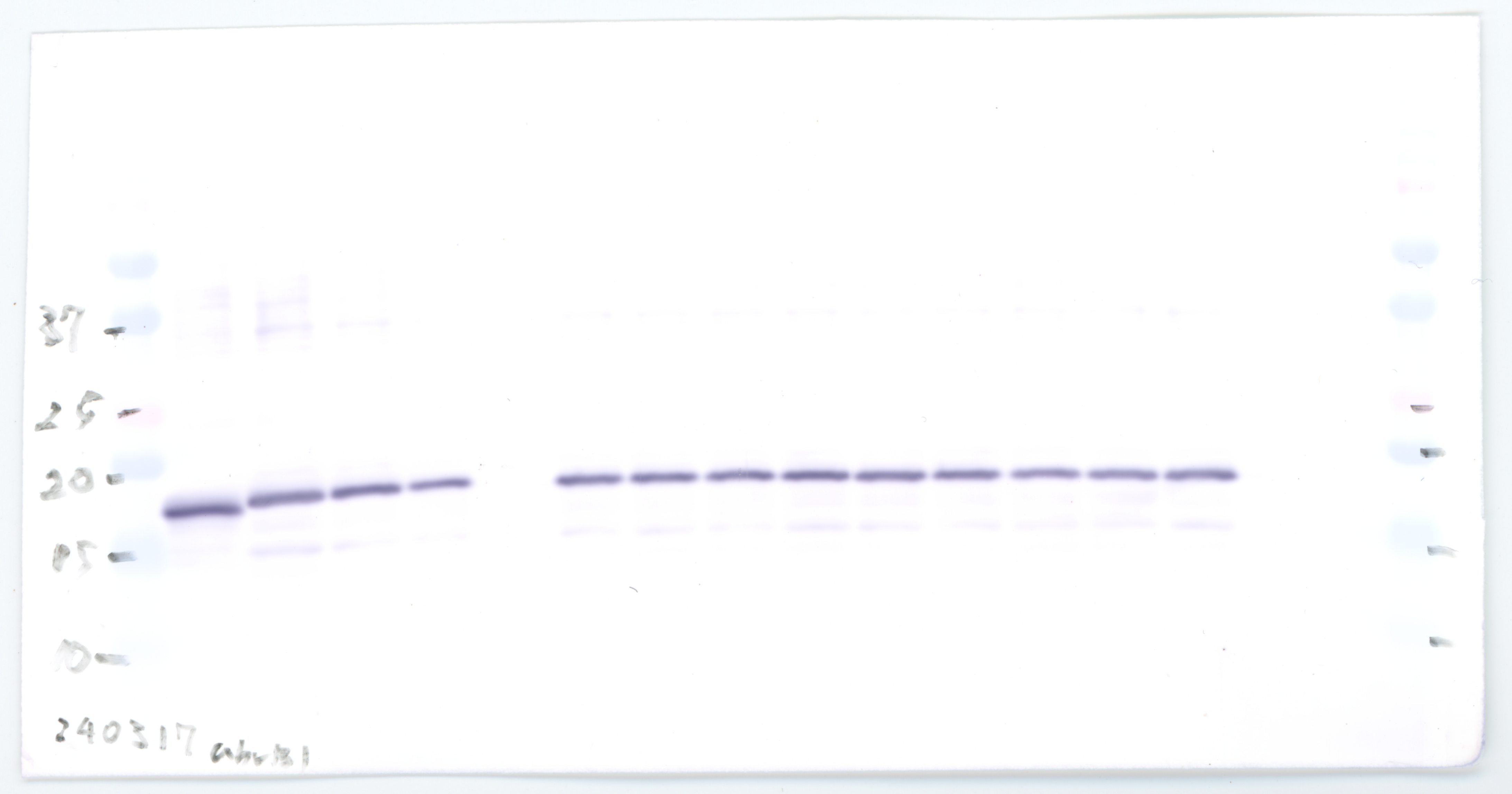

Supplement: Source data 1. [file elife-94245-data1.zip › original_images/Figure5-figure supplement 2.tif]

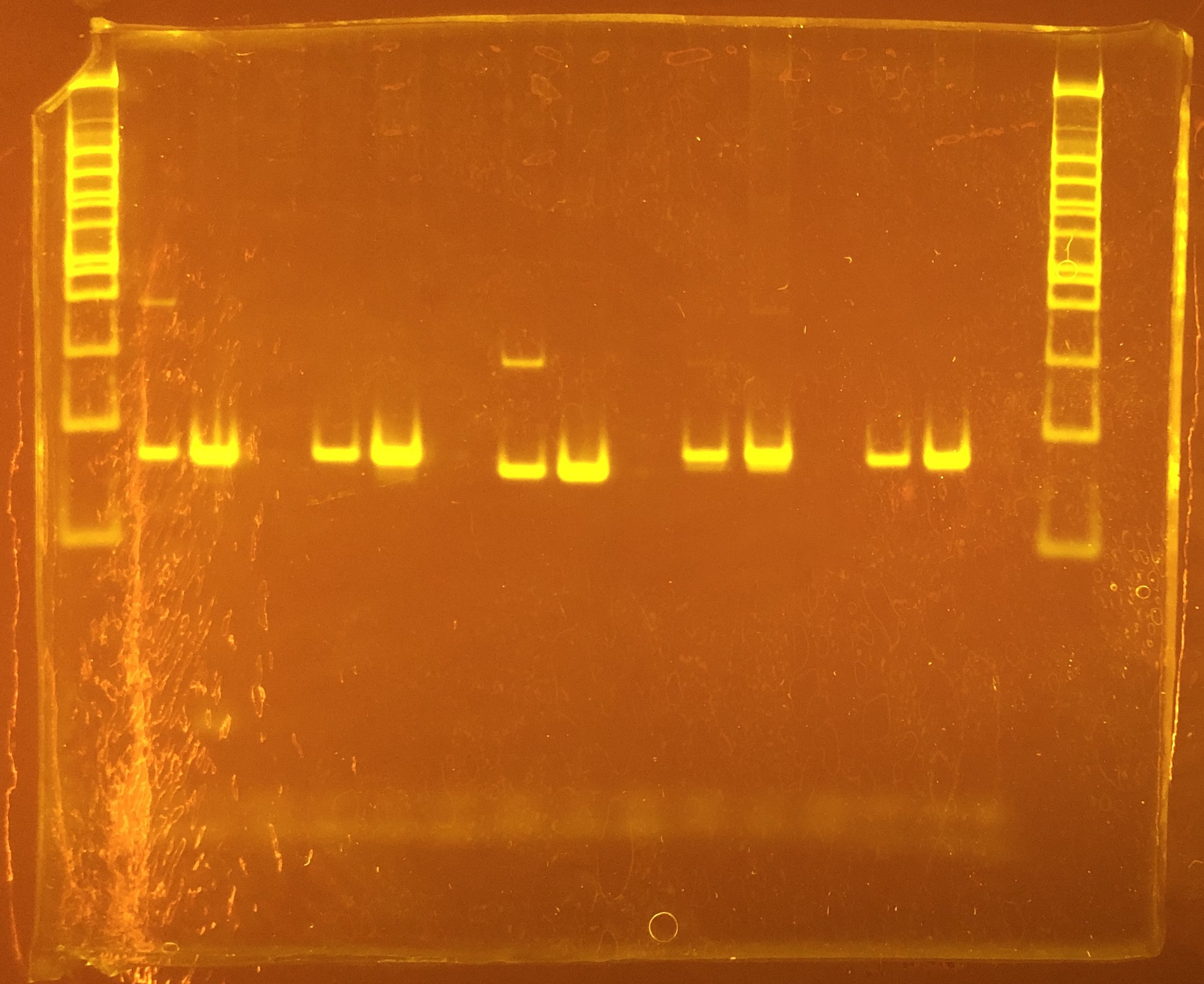

Supplement: Source data 1. [file elife-94245-data1.zip › original_images/Figure7-figure supplement 2/240317_3C_nifJ_chk_2.jpg]

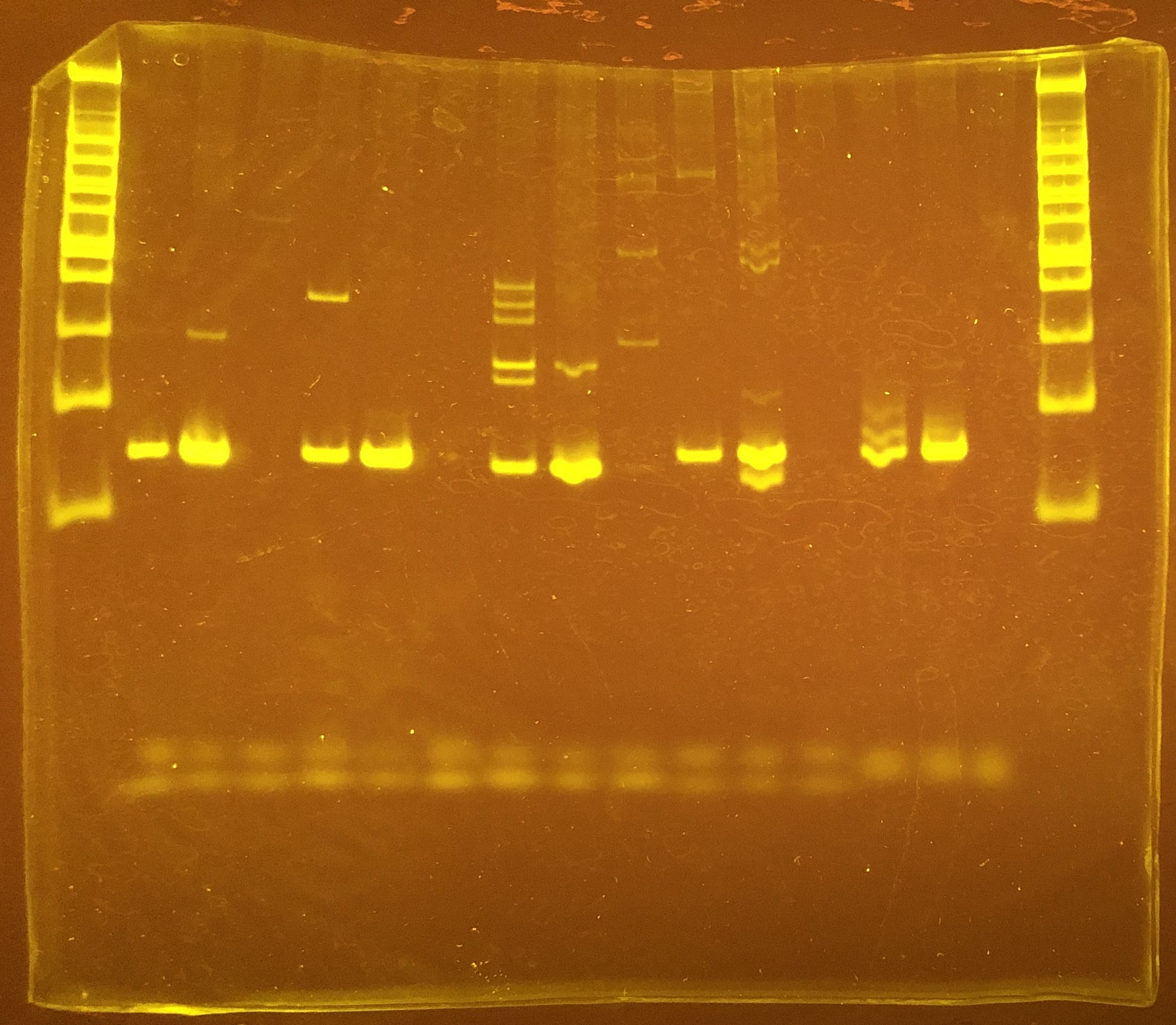

Supplement: Source data 1. [file elife-94245-data1.zip › original_images/Figure7-figure supplement 2/240317_3C_nifJ_chk_3.jpg]

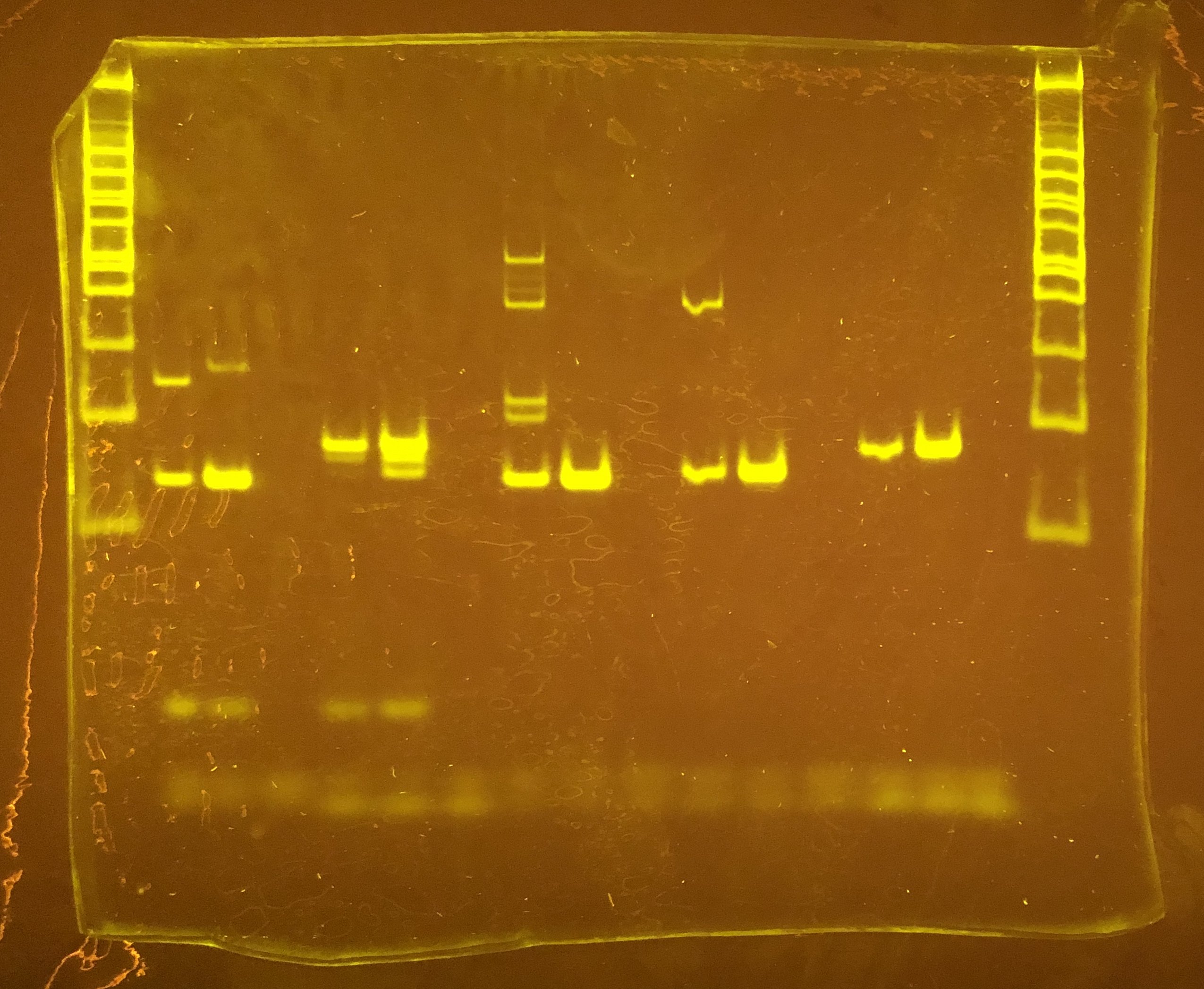

Supplement: Source data 1. [file elife-94245-data1.zip › original_images/Figure7-figure supplement 2/240317_3C_nifJ_chk_1.jpg]

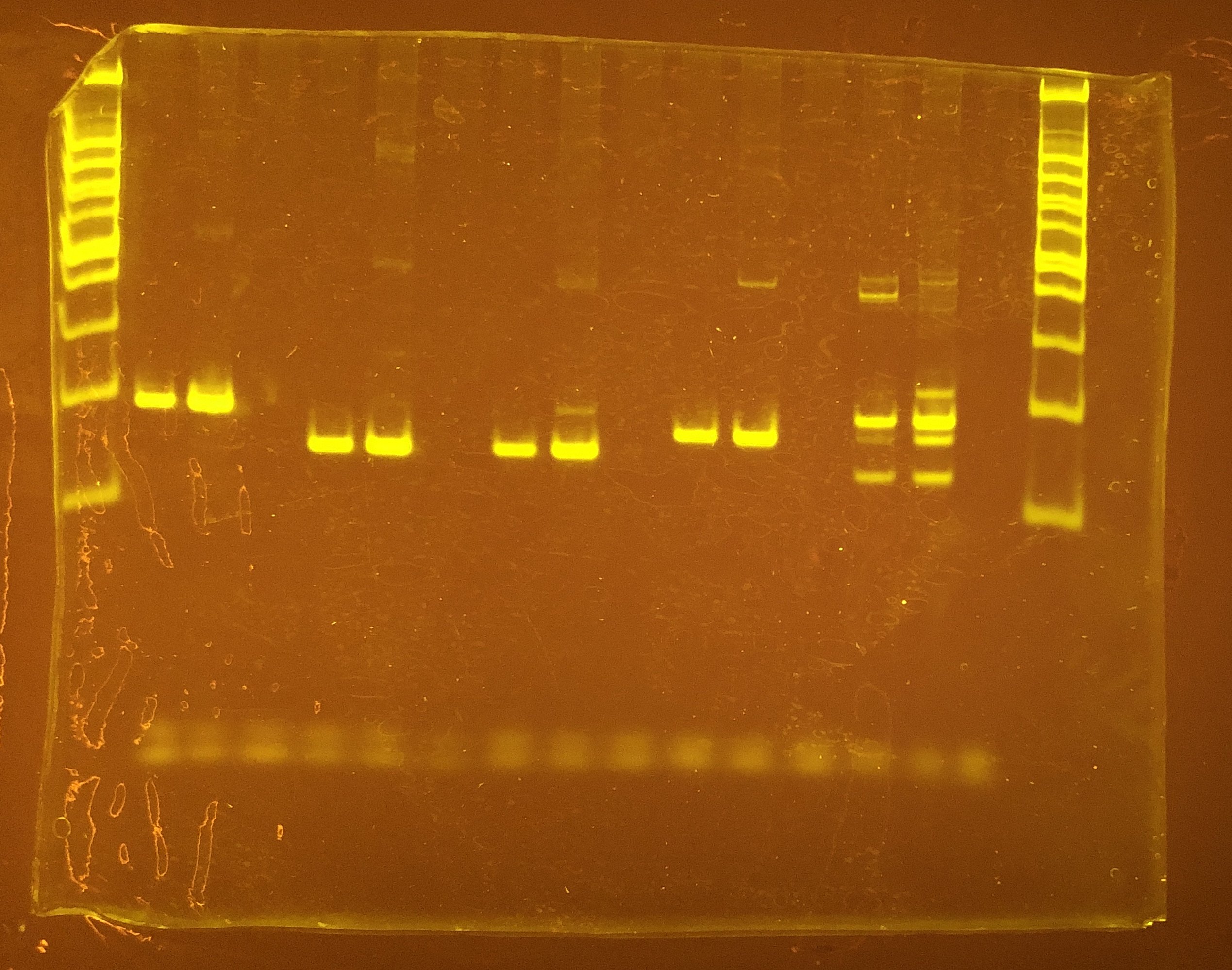

Supplement: Source data 1. [file elife-94245-data1.zip › original_images/Figure7-figure supplement 2/240317_3C_nifJ_chk_4.jpg]

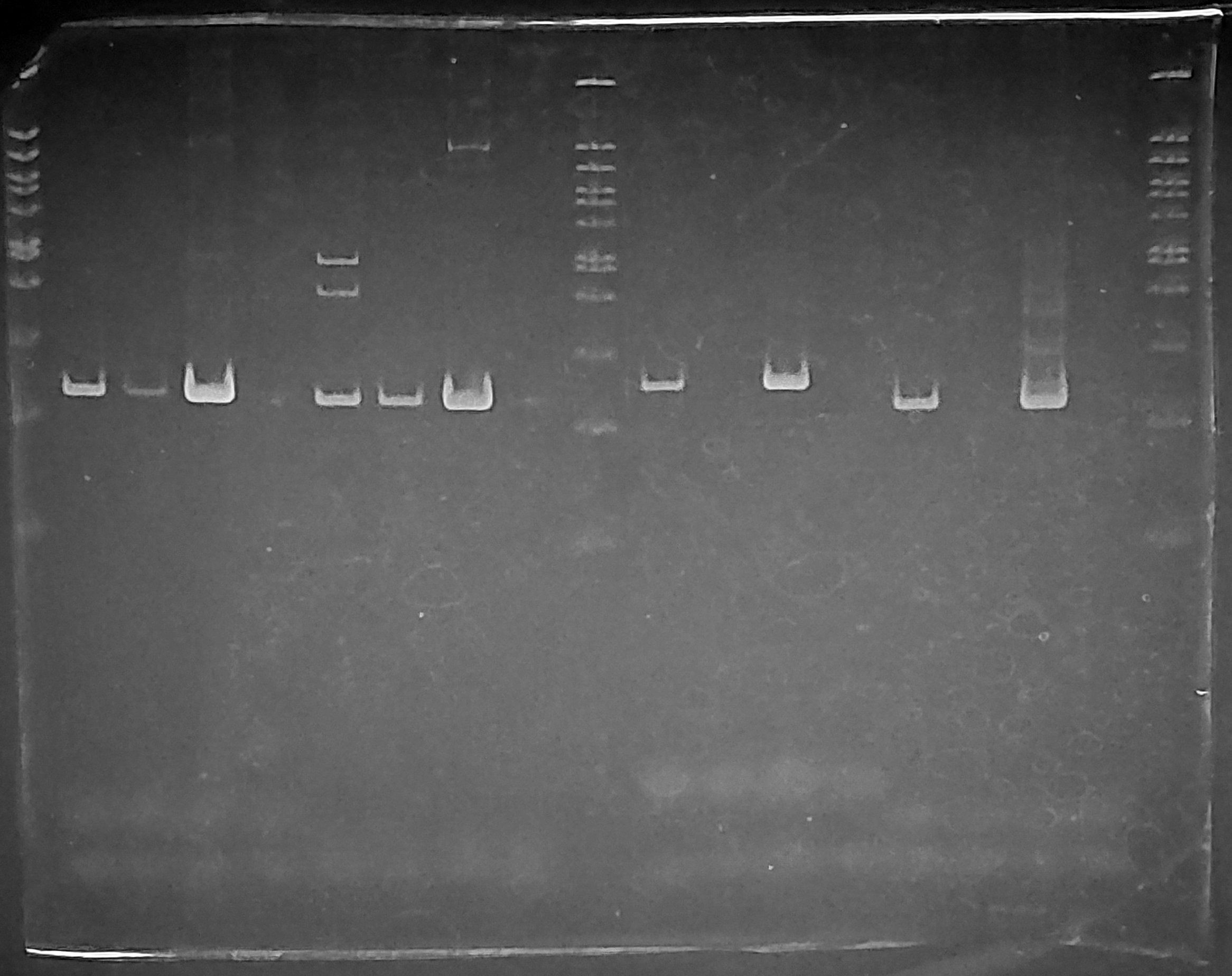

Supplement: Source data 1. [file elife-94245-data1.zip › original_images/Figure7-figure supplement 2/230720_3C_hox_chk3.jpg]

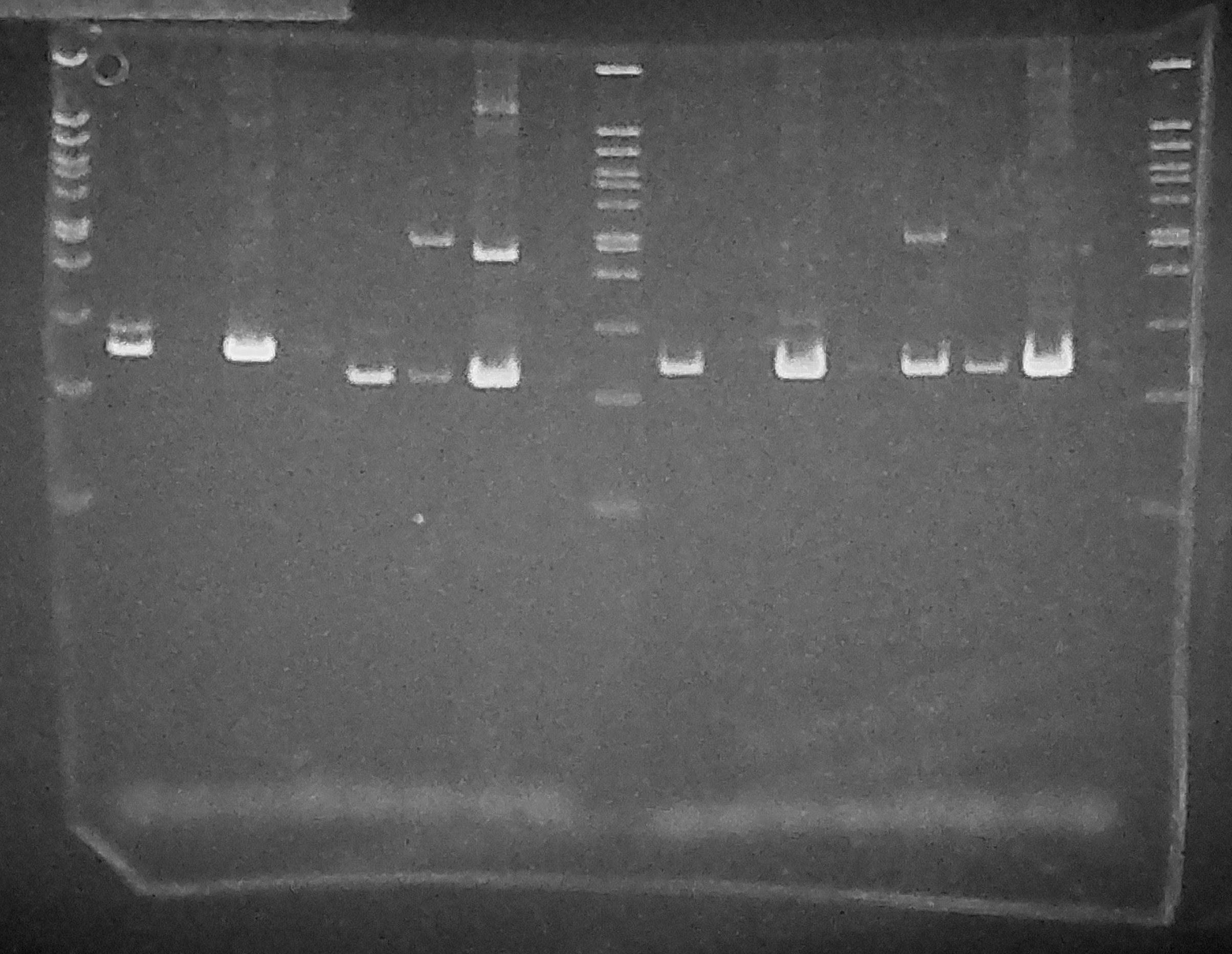

Supplement: Source data 1. [file elife-94245-data1.zip › original_images/Figure7-figure supplement 2/230720_3C_hox_chk2.jpg]

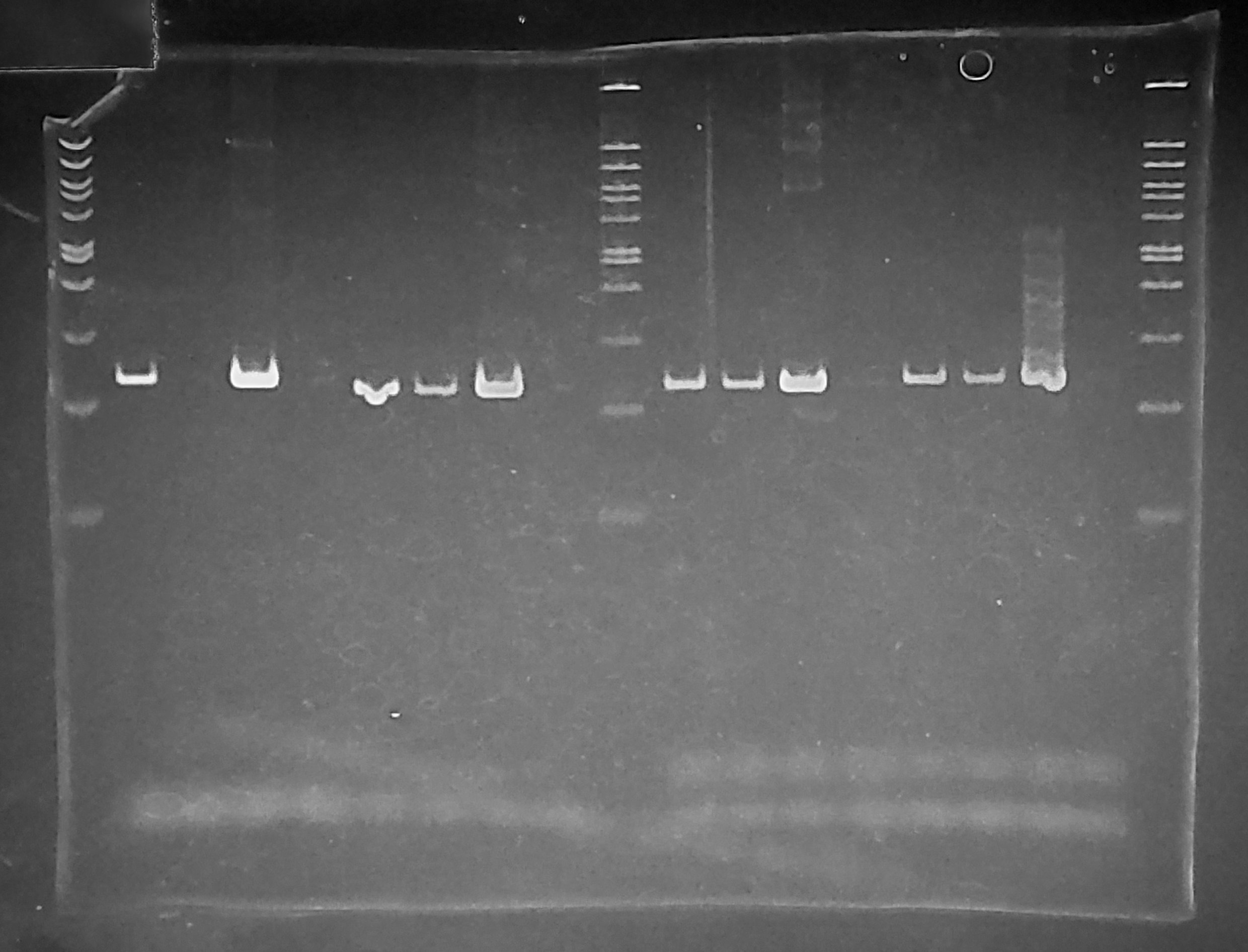

Supplement: Source data 1. [file elife-94245-data1.zip › original_images/Figure7-figure supplement 2/230720_3C_hox_chk1.jpg]

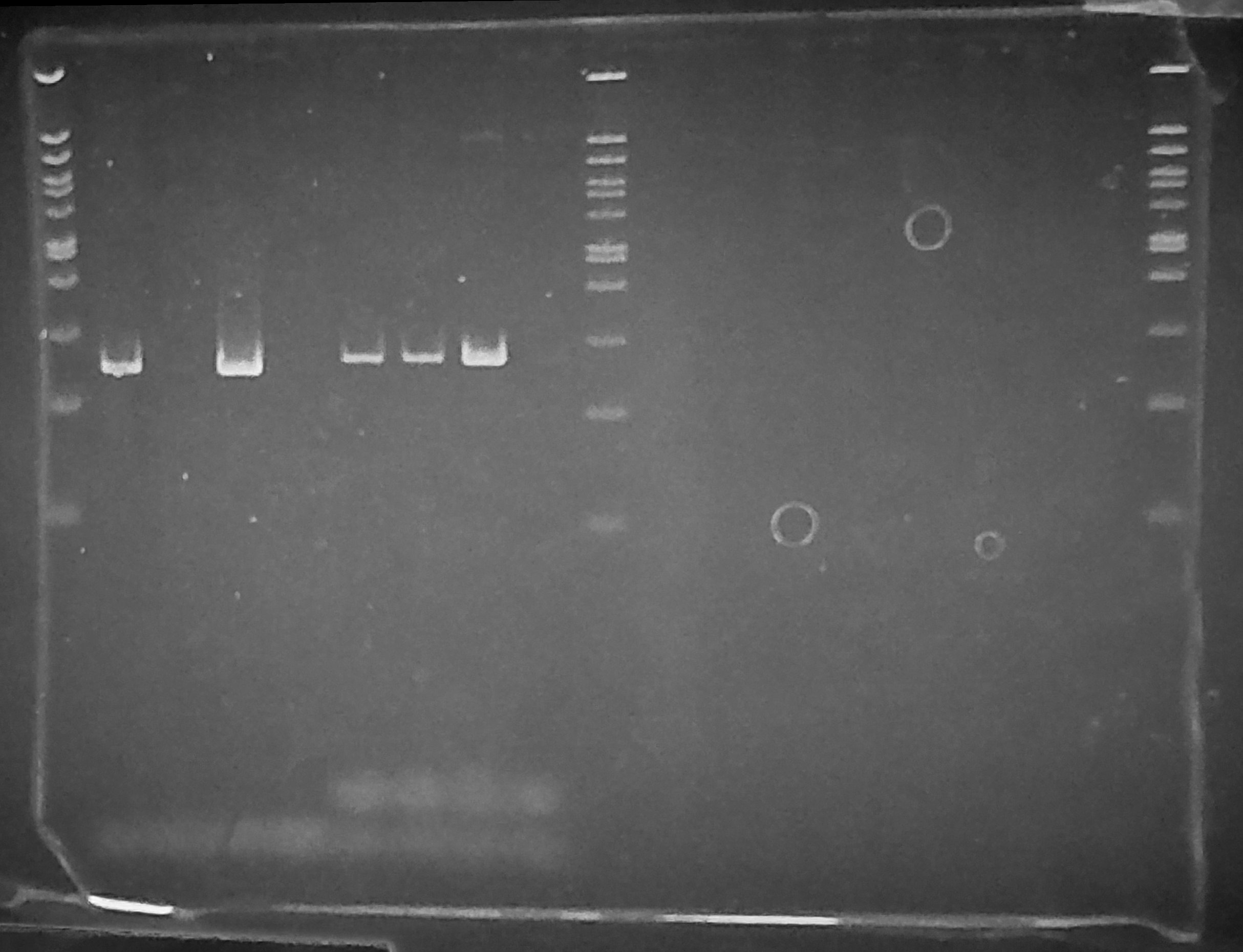

Supplement: Source data 1. [file elife-94245-data1.zip › original_images/Figure7-figure supplement 2/230720_3C_hox_chk4.jpg]
